# Modified NOTES technique for creation of an endoscopic gastrojejunostomy

**DOI:** 10.1055/a-2463-4015

**Published:** 2024-11-26

**Authors:** Natalie J. Wilson, Rahul Karna, Mohammad Bilal

**Affiliations:** 1 Department of Internal Medicine, University of Minnesota, Minneapolis, United States; 2 Division of Gastroenterology, Hepatology, and Nutrition, University of Minnesota, Minneapolis, United States; 3 Division of Gastroenterology and Hepatology, Minneapolis Veterans Affairs Medical Center, Minneapolis, United States

Endoscopic ultrasound-guided gastrojejunostomy (EUS-GJ) with a lumen-apposing metal stent (LAMS) relies on endosonographic visualization of the jejunum. In cases where jejunal visualization is not possible, natural orifice transluminal endoscopic surgery (NOTES) can offer an alternative technique for performing endoscopic gastrojejunostomy (EGJ).

A 70-year-old man with metastatic urothelial carcinoma presented with gastric outlet obstruction from tumor infiltration of the distal duodenum and jejunum. He was deemed a nonsurgical candidate and referred for enteral stenting or EUS-GJ. However, EUS-GJ was not feasible because the guidewire could not traverse the obstruction. Furthermore, the jejunum could not be visualized endosonographically for direct EUS-GJ. After multidisciplinary discussion, the decision was made to attempt EGJ using NOTES.

The peritoneal cavity was accessed from the stomach using a 19-gauge needle under EUS guidance and a guidewire was passed ( Video 1 ). The cautery feature of the LAMS catheter (20 × 10 mm Hot AXIOS; Boston Scientific; Marlborough, Massachusetts, USA) was used to create a tract in the gastric body. A dual-channel endoscope was advanced into the peritoneal cavity and the jejunum was identified. A needle-knife was used to make an incision in the jejunum and a guidewire was passed into the jejunum. The LAMS catheter was then advanced over the guidewire and the distal flange of the LAMS was deployed. The jejunum was retracted toward the stomach using grasping forceps and the proximal flange was subsequently deployed in the stomach under endoscopic visualization, establishing the EGJ ( Fig. 1 ). The patient was started on immunotherapy and had no recurrence of gastric outlet obstruction after 5 months of follow-up.

Modified natural orifice transluminal endoscopic surgery technique for creation of an endoscopic gastrojejunostomy.Video 1

**Fig. 1:**
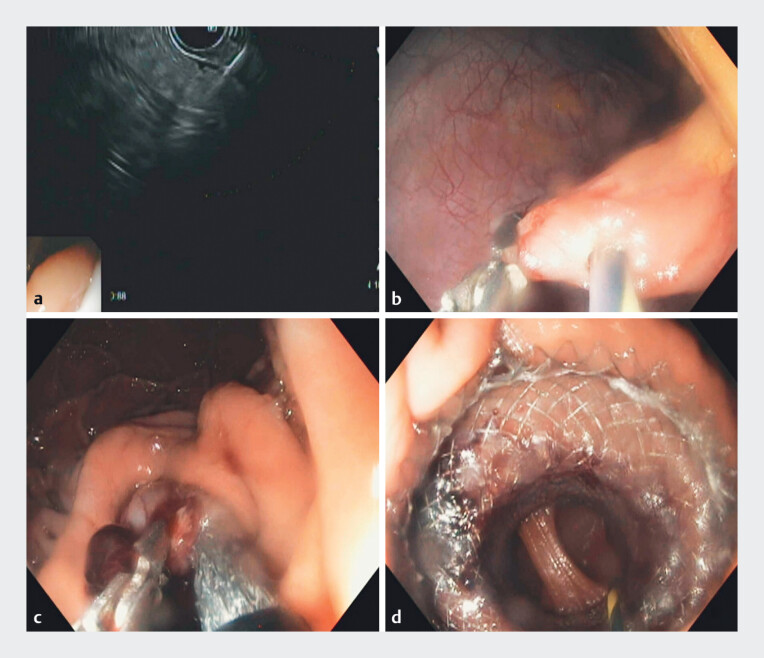
Endoscopic procedure. **a** Endoscopic ultrasound-guided puncture of the peritoneal cavity. **b** Using a dual-channel endoscope, the jejunum was held in place with grasping forceps while a guidewire was passed into the jejunum through a needle-knife incision. **c** The jejunum was retracted toward the stomach while keeping traction on the grasping forceps. **d** Endoscopic visualization of successful gastrojejunostomy creation.

In this case, we used the cautery function of the LAMS catheter to create the gastric tract, and subsequently the same catheter was used to deploy the LAMS across the EGJ. This method could be used if the diathermic cystotomes used in previous reports are not available 1 .

Endoscopy_UCTN_Code_TTT_1AS_2AB

## Notes

Endoscopy E-Videos https://eref.thieme.de/e-videos
*E-Videos* is an open access online section of the journal *Endoscopy* , reporting on interesting cases and new techniques in gastroenterological endoscopy. All papers include a high-quality video and are published with a Creative Commons CC-BY license. Endoscopy E-Videos qualify for HINARI discounts and waivers and eligibility is automatically checked during the submission process. We grant 100% waivers to articles whose corresponding authors are based in Group A countries and 50% waivers to those who are based in Group B countries as classified by Research4Life (see: https://www.research4life.org/access/eligibility/ ). This section has its own submission website at https://mc.manuscriptcentral.com/e-videos .
